# DnaJ homolog Hdj2 Facilitates Japanese Encephalitis Virus Replication

**DOI:** 10.1186/1743-422X-8-471

**Published:** 2011-10-14

**Authors:** Robert Yung-Liang Wang, Yu-Ru Huang, Ka-Man Chong, Chun-Yu Hung, Zhi-Long Ke, Ruey-Yi Chang

**Affiliations:** 1 Department of Biomedical Sciences, Chang Gung University; Tao Yuan 33302, Taiwan, ROC; 2 Research Center for Emerging Viral Infections, Chang Gung University, Tao Yuan 33302, Taiwan, ROC; 3 Institute of Biotechnology and Department of Life Science, National Dong Hwa University, Hualien 97401, Taiwan, ROC

**Keywords:** JEV, Hsp40, Hdj2, and RdRp

## Abstract

**Background:**

Japanese encephalitis virus (JEV) is a member of the mosquito-borne *Flaviviridae *family of viruses that causes human encephalitis. Upon infection of a new host, replication of viral RNA involves not only the viral RNA-dependent RNA polymerase (RdRp), but also host proteins. Host factors involved in JEV replication are not well characterized.

**Results:**

We identified Hdj2, a heat-shock protein 40 (Hsp40)/DnaJ homolog, from a mouse brain cDNA library interacting with JEV nonstructural protein 5 (NS5) encoding viral RdRp using yeast two-hybrid system. Specific interaction of Hdj2 with NS5 was confirmed by coimmunoprecipitation and colocalization in JEV-infected cells. Overexpression of Hdj2 in JEV-infected cells led to an increase of RNA synthesis, and the virus titer was elevated approximately 4.5- to 10-fold. Knocking down of Hdj2 by siRNA reduced the virus production significantly.

**Conclusions:**

We conclude that Hdj2 directly associates with JEV NS5 and facilitates viral replication. This study is the first to demonstrate Hdj2 involved in JEV replication, providing insight into a potential therapeutic target and cell-based vaccine development of JEV infection.

## Background

Japanese encephalitis virus (JEV), a mosquito-borne flavivirus, is a leading cause of encephalitis in Eastern and Southern Asia. The virus has a zoonotic transmission cycle, with swine serving as the readily available amplifying hosts for which infected mosquitoes transmit the virus to humans. There are three very effective JEV human vaccines that have been used and have reduced the impact of JEV infection in Asian countries. These are the Chinese-developed SA14-14-2 live-attenuated vaccine, the conventional suckling mouse-brain derived, formalin-inactivated vaccine, and the Vero cell derived, formalin-inactivated vaccine [1,2]. However, with no effective antiviral drugs available and high fatality rates in humans, JE remains a worldwide public health problem [3,4]. The JEV genome is a single-strand positive sense RNA, 10,976 nucleotides (nts) in length. The RNA strand contains a type I cap at its 5' end, and lacks a poly A tail at its 3' end. The genome encodes a large open reading frame (ORF) flanked by 5' and 3' untranslated regions (UTRs). The single ORF is translated to a polyprotein, which undergoes co- and post-translational processing into three structural proteins (C, prM, and E), and seven nonstructural proteins (NS1, NS2A, NS2B, NS3, NS4A, NS4B, and NS5) [5].

The largest viral protein, NS5, is a multifunctional protein. Although the structure and function of JEV NS5 has not been thoroughly investigated, a comparison of the deduced flavivirus amino acid sequences reveals several homologous regions that indicate similar NS5 function among the flaviviruses [6]. The N-terminal part of NS5 is a methyltransferase enzyme, and the NS5 C-terminal exhibits RNA-dependent RNA polymerase (RdRp) activity. The conserved GDD motif within the RdRp domain plays a major role in RNA amplification [7,8]. Although the replication of flaviviruses occurs in the cytoplasm, a number of studies report that 20% of the RdRp is resident within the nucleus [9,10]. The JEV NS5 protein contains a nuclear localization signal (NLS) responsible for protein translocation into the nucleus. Although the purpose of nuclear localization for viral protein remains unknown, it may facilitate viral replication, and could alter host metabolism upon JEV infection. In addition to viral replication, NS5 may also play a role in pathogenesis [11]. Recent advances in our understanding of many functional roles, and the elucidation of the NS5 structure of other flaviviruses such as Dengue virus and West Nile virus, has made NS5 an important target for antiflaviviral drug development [6,12].

Numerous studies have demonstrated that host factors participate in RNA viral replication. These host factors enhance viral RNA replication, RNA stability, genome translation, posttranslational modification, formation of the replication complex, and viral assembly [13]. To obtain more information regarding the interactions between host factors and the NS5 protein, we performed a yeast-two hybrid assay using a mouse-brain cDNA library. We identified several host factors, and further characterized Hdj2, a heat-shock protein 40 (Hsp40)/DnaJ homolog. Heat-shock proteins (Hsps) are cellular chaperons that normally facilitate cellular protein translation, folding, trafficking, and degradation [14]. Hsp40 is often referred to as a co-chaperon; it associates with Hsp70 and influences chaperon function [15,16]

In this study, we confirmed the interaction of Hdj2 with NS5 by coimmunoprecipitation (Co-IP), Western blotting, and immunofluorescence assay. Results from overexpression and silencing of the expression of Hdj2 protein in JEV-infected cells, indicate that host Hdj2 associates with JEV NS5 and facilitates viral replication.

## Results

### JEV NS5 protein interacts with Hdj2 *in vivo*

**Figure 1:**
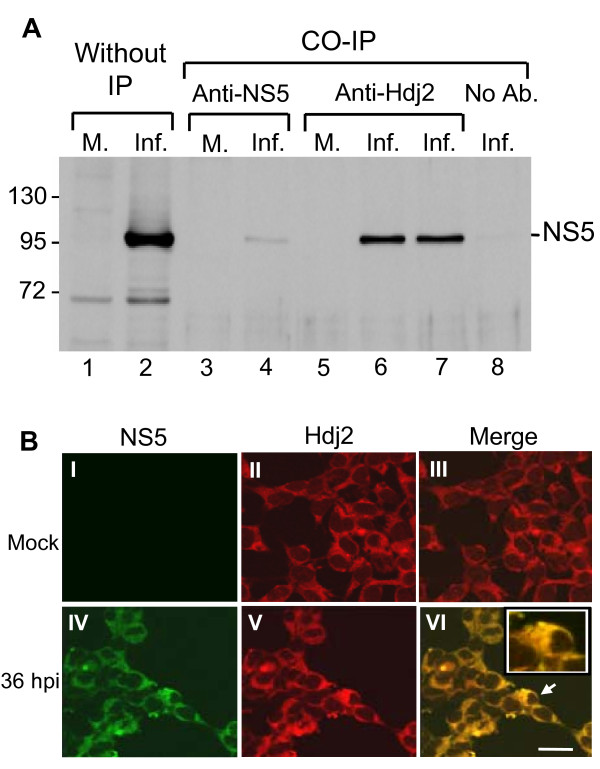
**JEV viral NS5 interacts with Hdj2 *in vivo***. A. HEK293 cells were mock-infected or infected with JEV and harvested at 48 h post-infection. Cell lysates were subjected to co-immunoprecipitation (IP) with antibodies against NS5 or anti-Hdj2, followed by Western blot analysis for the detection of NS5. Lanes 1 and 2, Mock- and JEV-infected cell lysates; lanes 3 and 4, Co-IP with anti-NS5 antibody; lanes 5-7, Co-IP with anti-Hdj2 antibody (0.5 μg and 1 μg of anti-Hdj2 antibody was used for lanes 6 and 7, respectively); lane 8, Co-IP with no added antibody. B. Mock- or JEV-infected HEK293 cells were stained by indirect immunofluorescence using rabbit anti-NS5 polyclonal antibodies (panel I, IV), and mouse anti-Hdj2 antibody (panel II, V) and detected by FITC-conjugated goat anti-rabbit and CY3-donkey anti-mouse antibody, respectively. Panels III and VI are merged images from two previous panels. The enlarged image of the selected area marked by an arrow is given in the upper right corner of panel VI. Cells were viewed at 1000 × magnification; a 20 μm scale bar is shown.

Hdj2 is among the candidates that interact with NS5 by yeast two-hybrid assay (described in Materials and Methods); thus, specificity of the interaction requires verification to avoid a false positive result. To this end, cell lysates from mock- and JEV-infected cells were harvested and subjected to immunoprecipitation with anti-Hdj2 antibody. The immunoprecipitates were characterized by Western blot analysis using anti-NS5 specific antibody. Figure 1A shows coimmunoprecipitation of NS5 with anti-Hdj2 antibody in JEV-infected cells (Figure 1A, lanes 6 and 7), but not in the mock-infected cells (Figure 1A, lane 5), indicating a specific interaction between host Hdj2 and the viral NS5 protein. By contrast, no NS5 was detected when coimmunoprecipitation without any antibody added to the JEV-infected lysates (Figure 1A, lane 8) or negative control [17]. These observations indicate that Hdj2 interacts with NS5 in JEV-infected cells.

### Colocalization of Hdj2 and NS5 in JEV-infected cells

To investigate the intracellular distribution of the NS5 and Hdj2 upon JEV infection, we examined the localization of these proteins using confocal microscopy of the mock- and JEV-infected cells. Dual-labeled immunofluorescence staining was conducted using rabbit anti-NS5 serum and mouse anti-Hdj2 serum as primary antibodies in combination with FITC-conjugated anti-rabbit and Cy3-conjugated anti-mouse secondary antibodies. The expression of viral NS5 protein was detected predominantly in the cytoplasm of infected cells (Figure 1B, panel IV), but not in the mock-infected cells (Figure 1B, panel I). The constitutive expression of Hdj2 was detected in both mock-infected and infected cells (Figure 1B, panels II & V). Upon JEV infection, the images of dual-labeled immunofluorescence showed yellow coloration (Figure 1B, panel VI), indicating that NS5 and Hdj2 proteins colocalize in the cytoplasm of JEV-infected cells.

### Overexpression of recombinant Hdj2 protein facilitates JEV replication

**Figure 2:**
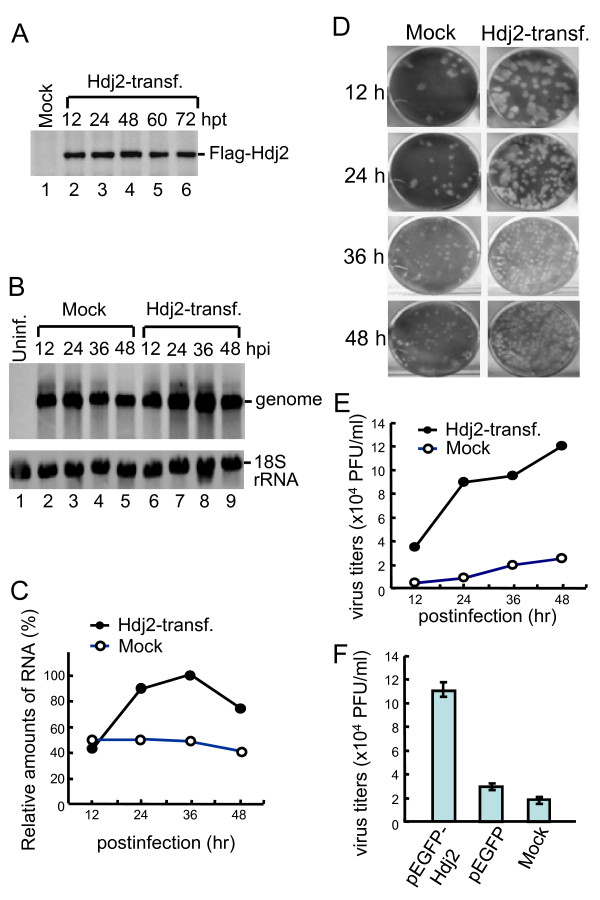
**Overexpression of Hdj2 facilitates virus replication**. A. HEK293T cells were mock-transfected or transfected with Flag-Hdj2 expressing plasmid. The cells were harvested at the indicated hour post-transfection (hpt), and subjected to Western blotting analysis using anti-Flag antibody for detection of Flag-Hdj2 expression. B. Transfected cells were infected with JEV. The total RNA and supernatant were harvested from mock or Hdj2-transfected at the indicated hour post-infection (hpi) and subjected to Northern analysis, or virus titer determination, respectively. The RNA was probed with a DIG-labeled oligonucleotide detecting nt 10950 to nt 10976 in the 3' UTR and an oligonucleotide probe detecting 18S rRNA. C. The amounts of RNA genome were normalized to that present in the 18S rRNA, and the relative amounts were plotted. D and E. The virus titers from each time point were calculated and plotted. F. JE virus titer was measured at 48 h post-infection of pEGFP-Hdj2, pEGFP, or mock-transfected cells. Error bars indicate the standard deviations of results from three independent experiments.

To test whether Hdj2 facilitates JEV replication, the Hdj2 gene was cloned into p3XFLAG-Myc-CMV-26 expression vector (see Materials and Methods) and transfected into HEK293T cells. High expression levels of Flag-tag Hdj2 were observed as early as at 12 h post-transfection (Figure 2A, lane 2), and were maintained to 72 h post-transfection (Figure 2A, lanes 3-6). Thus, infection of JEV was performed at 12 h post-transfection of the overexpressed Flag-tag Hdj2, followed by total RNA extraction at 12, 24, 36, and 48 h post-infection for the detection of viral RNA. The results showed that JEV RNA synthesis increased 2- to 4-fold in the Hdj2 transfected cells, compared to mock-transfected cells (Figure 2B &2C). Supernatant containing newly released virus particles was collected at the same time points as indicated above to determine the virus titer. The titer increased 4.5- to 10-fold in the Hdj2 transfected cells, compared to mock-transfected cells (Figure 2D &2E). The virus titer also increased in cells transfected with Hdj2 as a fusion protein with green fluorescence protein (pEGFP-Hdj2), but not in the pEGFP-transfected, or mock-transfected cells (Figure 2F). Overall, these results indicate that overexpression of Hdj2 protein facilitates JEV replication.

### Silencing Hdj2 impairs JEV replication

**Figure 3:**
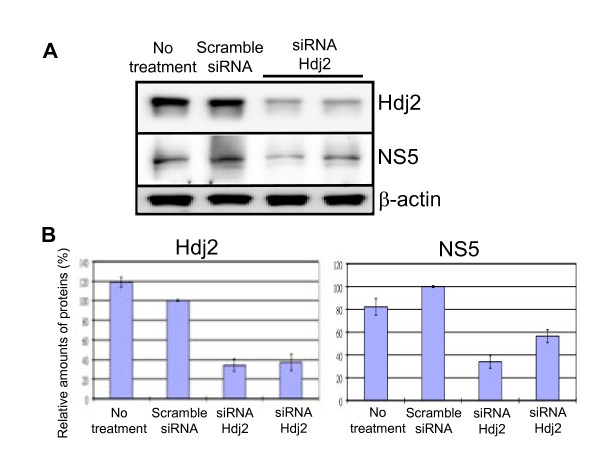
**Silencing Hdj2 using siRNA decreases JEV NS5 production**. HEK293 cells were transfected with siRNA against Hdj2, or scrambled siRNA (negative control, see the description in the Materials and Methods). A. The down-regulation of Hdj2 was determined by Western blot analysis using antibodies specific to Hdj2. The transfected cells were then infected with JEV. The cell lysates were collected at 24 h post-infection and subjected to western blot analysis using anti-Hdj2, anti-NS5, and anti β-actin specific antibodies. B. Quantitative measurement of the indicated band intensities of Hdj2 and NS5 from JEV-infected scrambled siRNA treated cells, or cells treated with siRNA against Hdj2. Results were derived from three independent experiments.

To further investigate the role of Hdj2 in JEV replication, we treated HEK293 cells with Hdj2 siRNA and negative control siRNA prior to JEV infection. Western blot analysis showed that Hdj2 levels in the knockdown cells were reduced to approximately 40% of those observed in control cells (Figure 3A). The cells were then infected with JEV. At 24 h post-infection, the NS5 expression level was reduced by approximately 50% in Hdj2-silenced cells in comparison with the untreated or scrambled siRNA treated cells (Figure 3B). Furthermore, Hdj2-silenced cells also reduced the virus titer (data not shown). This result supports the hypothesis that Hdj2 is essential for JEV replication.

## Discussion

Dissecting the host factors involved in viral replication helps understand the molecular mechanism of viral pathogenesis. In this study, we show for the first time that JEV NS5 interacts with one member of the heat-shock protein family, Hdj2, which we identified by yeast two-hybrid screening, and further confirmed by immunoprecipitation and colocalization studies (Figure 1).

Cellular heat-shock proteins are chaperon molecules, known to participate in a variety of cellular processes such as protein folding, protein translocation, and protein degradation [18]. Hdj2 belongs to the type I DnaJ (Hsp40) family [19]. Hsp40 binds to unfolded proteins, interacts with Hsp70 as a cochaperon, and stimulates ATP hydrolysis [20]. The type I DnaJ family is defined by the presence of an N-terminal J domain, followed by a glycine and phenylalanine-rich domain, a cysteine-rich zinc finger domain, and a variable C-terminal domain. A conserved histidine-proline-aspartic acid tripeptide motif (HPD motif) in the J domain is critical for accelerating Hsp70 ATPase activity [21,22]. Additionally, Hdj2 is a multifunction protein that includes (i) facilitating androgen receptors bound to androgen through the zinc finger domain [23]; (ii) regulation of mitochondrial protein import [24,25]; and (iii) cooperation with Hsp70 to suppress protein aggregation and apoptosis [26,27].

Although Hsp70 involvement at different stages of the viral life cycle, including entry, replication, gene expression, and encapsidation has been extensively studied, there are relatively few reports of Hsp40 involvement in the viral life cycle [15]. Hsp40 may ensure the correct conformation of the viral replication complex, or may interact with viral proteins, and often facilitates viral replication [28,29]. Hsp40 (Ydj1P) participated in viral RdRp folding in *Brome mosaic virus*-infected cells, and facilitated viral negative-strand RNA synthesis [30]. DnaJ homologs play a role in suppressing virus-induced apoptosis [31], and furthermore, DnaJ homologs suppress antiviral processes by regulating the activities of other cellular proteins. In Influenza virus-infected cells, P58^IPK ^(DnaJ homolog) phosphorylation of PKR was inhibited, blocking the antiviral pathway [32].

Flavivirus infection involves the synthesis of large numbers of viral proteins, which induce ER stress, causing an unfolded protein response in infected cells, leading to cell apoptosis [33,34]. JEV NS5 proteins, as with other flaviviruses, were shown to be interferon (IFN) antagonists [35,36]. NS5 blocks the IFN-stimulated JAK/Stat pathway, and evades IFN-induced responses. Since NS5 interacts with Hdj2, it is intriguing to know whether this interaction helps NS5 interfere with IFN or other antiviral mechanisms. Although it is not known at what stage Hsp40 plays a role in JEV infection, our data clearly indicate that overexpression of Hdj2 enhances viral gene expression and viral production (Figure 2), and that the inhibition of Hdj2 expression reduces viral gene expression (Figure 3). These results suggest that JEV recruits the host heat-shock proteins to facilitate its replication. We postulate that mechanism(s) to enhance viral replication could (i) facilitate viral RNA replication, (ii) suppress apoptosis of the host cells, (iii) block host cell antiviral mechanisms, or (iv) may operate by other unidentified mechanisms. Natural hosts of JEV include mosquitoes, humans, waterfowls, and pigs. The body temperatures of these species range from room temperature to greater than 40°C. It is therefore likely that the virus recruits a host heat-shock protein to maintain proper conformation of the RdRp for the replication of viral genome at different temperatures. Hsp70 has been reported as a possible receptor for JEV infection [37,38]. Whether the interaction between Hsp40 and Hsp70 either directly or indirectly plays a role in virus production remains to be determined. Recently, Yi et al. reported that overexpression of DNAJC14, a member of Hsp40 chaperone proteins, inhibited the viral replication of various *Flaviviridae *family members [39]. However, silencing DNAJC14 also inhibited replication of the yellow fever virus, indicating that the host Hsp40 chaperone may be involved in more than one stage of the flavivirus life cycle.

Vaccination is the most effective method of preventing JE infection in humans. The currently used vaccine includes a mouse brain-derived, formalin-inactivated JEV or a new inactivated, Vero cell culture-derived JE vaccine [40]. It has been showed that there is a significant risk in using the inactivated mouse brain-derived vaccine, particularly regarding neurological and allergic reactions [41]. Thus, the cell culture-based vaccine is a better alternative than the mouse-brain derived vaccine. Because of the high cost of tissue culture, our finding that overexpression of Hdj2 significantly increases the viral titer, could serve as a basis for further improvements of cell culture-based vaccine.

## Conclusions

This is the first report of host heat-shock protein being directly associated with NS5, and facilitating virus replication. Identification of the interacting domains in NS5 and Hdj2 proteins is necessary to understand protein-protein interactions. Knowledge of the structures of these interacting domains would be useful for antiviral drug design and for the improving virus yield in the tissue culture-based live attenuated or inactivated vaccine.

## Materials and methods

### Cells and viruses

We used the JEV RP-9 strain in this study [42]. Baby hamster kidney (BHK-21) cells were used for JEV propagation and plaque assay. BHK-21 cells were grown in RPMI-1640 medium (Invitrogen) supplemented with 2% fetal bovine serum (FBS) (Invitrogen) at 37°C. Human embryonic kidney (HEK293 and HEK293T) cells were grown in DMEM medium (Invitrogen) containing 10% FBS.

### Yeast two-hybrid screening

The bait plasmid, pGBKT7-NS5, was constructed by cloning the PCR amplified, full-length JEV NS5 gene, into the *Bam*HI site of pGBKT7 (Clontech) in frame with the GAL4 DNA binding domain and transformed into *Saccharomyces cerevisiae *strain Y190. A Matchmaker pretransformed mouse brain cDNA library (Clontech) was screened via yeast mating by incubating 1 ml of the pretransformed library (5 × 10^6 ^CFU) with the bait-transformed Y190 strain, according to the manufacturer's directions. The entire mating culture was plated onto -His/-Leu/-Trp synthetic defined medium containing 25 mM 3-amino 1, 2,4-triazole (3-AT) and incubated at 30°C for 3 to 7 days. Colonies were picked, restreaked onto fresh selective medium, and were subjected to a LacZ colony-lift filter assay. Plasmid DNA was isolated from clones positive for β-galactosidase activity. Sequence analysis revealed that one of the selective positive clones is DnaJ homolog.

### Construction of plasmid

The p3XFLAG-Hdj2 plasmid was constructed by cloning the 1191-bp fragment, amplified from HEK293 cell cDNA, with Hdj2(+)1BglII (5'-gaagatctgATGGTGAAAGAAACAACTTA-3') and Hdj2(-)1191XbaI (5'-gctctgagGGTCTGACACTGAACA-3') primers, into p3XFLAG-Myc-CMV-26 expression vector (Sigma-Aldrich). The Hdj2 gene was also subcloned into pEGFP-C1 (Clontech) to form pEGFP-Hdj2. Sequences of the construct were confirmed by sequencing.

### Transfection and Northern blot analysis

Transfection was performed using Lipofectamine 2000 in OptiMEM according to the manufacturer's protocol (Invitrogen). Briefly, HEK293T cells in 60-mm plates at 50 to 60% confluence were transfected with 4 μg of p3XFLAG-Hdj2 (or pEGFP-Hdj2) plasmid DNA or mock transfected with H_2_O or vector alone. At 12 h post-transfection, cells were infected with JEV at a multiplicity of infection (MOI) of one, by incubating cells with inoculum at 37°C for 1 h, and refeeding with a growth medium until cytoplasmic RNA extraction was performed at 12, 24, 36, and 48 h post-infection. The supernatant was collected at the same time point as shown above, and titrated for infectious virus, as described [43]. Total RNA was extracted with REzolTM C&T reagent (Protech), and Northern analyses were conducted, as described [44].

### Coimmunoprecipitation assay

HEK293 cells (approximately 1 × 10^7 ^cells) were uninfected or infected with JEV RP9 at an MOI value of 0.1. At 48 h post-infection, cells were harvested, immunoprecipitated, and Western blotted as described elsewhere [17]. Briefly, cell lysates were incubated on ice for 10 min, and cell debris was removed by centrifugation. One milligram of whole cell extract per reaction was pre-cleaned with 20 μl protein G-plus agarose beads (Santa Cruz) at 4°C for 1 h with rotation. The clarified supernatants were incubated with anti-NS5 polyclonal antiserum prepared in rabbits [45] or mouse anti-Hdj2 monoclonal antibody (USBiological) at 4°C overnight. Prewashed protein G-plus agarose beads were added to the mixture, and incubated at 4°C for 1 h, and the immune complexes were washed four times with RIPA buffer. The final precipitate was boiled in a protein loading buffer for 5 min and eluted on SDS-10% PAGE for Western blot analysis.

### Immunofluorescence staining assay

HEK293T cells were seeded on coverslips (15 mm diameter) on 24-well tissue culture plates and incubated overnight. Cells were infected with JEV at an MOI of 0.1. At 36 h post-infection, cells were washed three times with cold phosphate-buffered saline (PBS), followed by fixation with ice-cold methanol. After washing with PBS and blocking with skimmed milk, samples were incubated with the rabbit anti-NS5 polyclonal antibody and mouse anti-Hdj2 monoclonal antibody (Jackson) for 1 h at 37°C. After five washes with PBS, the cells were incubated with FITC-conjugated goat anti-rabbit and CY3-conjugated donkey anti-mouse secondary antibodies (Jackson). After a further five washes with PBS, the coverslips were mounted and examined by confocal laser scanning microscopy (Leica TCS SL).

### siRNA transfection assay

Hdj2 siRNA (5'-CGUCAUCACCUCUCAUCCA-3') was designed and synthesized by Sigma Aldrich. Scrambled siRNA (medium GC of StealthTM RNAi) was used as a negative control (Invitrogen). The siRNA transfection was conducted using Lipofectamine RNAiMAX (Invitrogen) according to manufacturer's instruction. Briefly, 10 μl of Lipofectamine RNAiMAX and 180 μl of siRNA (at a final concentration 30 nM) were mixed with 1 ml OptiMEM (Invitrogen), followed by incubation for 20 min at room temperature. The mixture was then added with 5 ml cell growth medium and transfected into HEK293 cells for 2 days. The transfected cells were infected with JEV at an MOI value of 1. At 24 h post-infection, the supernatant was collected for quantitation of the virus titer, and the cell lysates were harvested for Western blot analysis using anti-Hdj2 (USBiological), anti-NS5, or anti-β-actin (Sigma) antibodies. Quantification of the intensity of bands on the Western blot has been done using the ImageJ gel analysis as described at http://lukemiller.org/index.php/2010/11/analyzing-gels-and-western-blots-with-image-j/.

## List of abbreviations

DnaJ: J-domain-containing cochaperon initially identified in *E. coli*; Hdj2: type I Hsp40; Hsp: heat shock protein; JEV: Japanese encephalitis virus; MOI: multiplicity of infection; NS5: nonstructural protein 5; RdRp: RNA-dependent RNA polymerase.

## Competing interests

The authors declare that they have no competing interests.

## Authors' contributions

RYW and YRH participated in the design of RNAi experiment and helped to draft the manuscript. YRH confirmed the interaction. KMC and ZLK carried out the overexpression experiments. CYH performed the knockdown experiments. RYC conceived of the study, participated in its design and coordination, and finalized the manuscript in its final form. All authors read and approved the final manuscript.
